# Demonstration of Container Effects on Recognition Process of Liquids Using a Ring-Resonator Measurement Method

**DOI:** 10.1038/s41598-019-49102-3

**Published:** 2019-08-29

**Authors:** Turgut Ozturk

**Affiliations:** Department of Electrical-Electronics Engineering, Bursa Technical University, Bursa, Turkey

**Keywords:** Electrical and electronic engineering, Characterization and analytical techniques, Electronic structure

## Abstract

Before separating or classifying hazardous/unsafe liquids, a system must be identified which can best measure certain liquids. The preferred measurement system can be useful, quick response, rapid measurement and so on. However, these features are not sufficient to classify the liquids in different containers. Especially at security points, containers of liquids carried by people may be different. Therefore, it has been investigated whether classification can be made by using the response of different containers to electromagnetic radiation. When the ring resonator measurement method is used in the 1–1.4 GHz frequency range, a successful separation process is carried out, even if the containers were made of different materials. Nevertheless, a well-known k-means algorithm has been used to analyze the measurement results of selected liquids.

## Introduction

Various methods can be tried to reduce the effects of dangerous materials and improve ways of protection. In the crowded factory, airport and shopping centers, security and safety precaution should be much higher. Where there is a lot of people, explosive and illegal materials are more likely to be carried by people. For this reason, a new method for the identification of hazardous materials should be presented where manual and visual search cannot be sufficient. Microwave spectroscopy systems, which may be an alternative method, have been performed in many application areas such as security or military. Free space measurement (FSM) method especially offers the possibility of especially non-destructive and non-contact measurements, characterization of solid-liquid-powder materials, measuring solid materials except very small ones, and without preparing sample^1,2^.

Material characterization process is a necessary stage in many applications. Nowadays, many measurement techniques are available to perform this goal and they are preferred according to the possibilities they provide. Microwave spectroscopy systems can be applied for specific studies to determine the fat content in milk, water pollution in lubricating oil, distinguish the materials as safe or unsafe, and classification of liquids. If the rate of water contamination in the oil rate is followed properly, it will be possible to reduce the system failures and the additional costs that may arise afterwards. It can be characterized using properties of a liquid such as permittivity, and S-parameters. For this, it is enough to collect the necessary information by measurement method without needing laboratory analysis. For the determination of quality of a new liquid-based product, the impact of many parameterizations must be considered. Trying to analyze each parameter separately for each liquid is not a preferred way in terms of both time and cost. Knowing many chemical methods may not be enough to facilitate this process. However, the use of the ring resonator method (RRM), which is one of the microwave spectroscopy systems that can produce a faster and more practical solution, can play an important role in characterizing and classification of liquids. The information needed here is to measure the change in the transmission parameter (*S*_21_)^3–6^.

The measuring processes of liquids were done in previous studies by performing different spectroscopies. The permittivity of liquids was extracted by using transmission line technique in different frequency bands. The extracted values also verified by Debye’s method in successfully^7^. The similar system to this one (in free space, transmission line) was presented between 5–40 GHz^8^. A new technology, which is substrate integrated waveguide - SIW based on resonant cavity, was proposed and performed for electrical characterization of liquids^9^. Alternatively, the reflectance and transmittance values of liquids were determined by using low-lost spectrometer^10^. The various liquids in the bag or on the human can be identified and classified using a multivariate data analysis algorithm, depending on the scattering parameters (dB) obtained with the microwave spectroscopy system. The important thing is here to determine the interaction the liquids under electromagnetic field at microwave frequencies. The complex permittivity was used to determine the liquid properties in previous studies^11^. Furthermore, dielectric properties of liquids have been obtained using measurement systems such as Hilbert spectroscopy and Josephson spectrometer, which are dual-mode and open-cavity resonators, respectively^12,13^. In addition, the nuclear magnetic resonance were performed to detect the unsafe liquids^14^. The measurement results of the microwave spectroscopy system have been analyzed by k-means algorithm, taking advantage of the difference in the reaction of the samples to electromagnetic radiation at microwave frequencies, unlike these studies.

Actually, the classification process is not a new field, such that a lot of studies have been performed to can identify and classify easily the plant leaves and diseases^15–17^. In addition, the algorithms of classification and regression trees have been tried to estimate material properties and behavior^18^. In fact, clustering and classification processes have emerged for solving many application problems such as vector quantization, data mining, pattern recognition and classification^19^. For this purpose, many classification algorithms can be proposed such as Self Organizing Maps (SOM), K-means, and Principal Component Analysis (PCA)^20,21^. Besides, Genetic Algorithm has been used for materials design and processing^22^. The effects of changes in viscosity, conductivity and permittivity parameters of each sample on the recognition of liquids were investigated. K-means, Self-Organizing Maps (SOM) and Principle Component Analysis (PCA) algorithms were used to classify and determine the unsafe liquids^4^. PCA technique was used for analysis and classification process of bulk metallic glasses materials as a multivariate data analysis method^23^. Support Vector Machine (SVM) method was suggested for material phase classification and has been performed successfully with Otsu-based approach^24^. In a different study, the classification and quantification process of microstructural constituents were investigated using SVM and the parameters affecting to the classification were tested^25^.

The classification of various liquids was successfully performed, in this study. The results showed that the illicit or explosive liquids can be distinguished using proposed model. Hence, the sudden reaction of samples under electromagnetic radiation is used to ease the classification process by statistical algorithms. When the sudden change in this radiation response of samples combines with the high-level performance of the classification algorithms, the expected output appears more clearly. In order to reduce the probability of error, the measurements were repeated for each liquid and the most accurate results were obtained. Thus, it is aimed to reduce the cost of the identification process, to shorten the measurement and duration and to make the definition of materials better.

## Ring-Resonator Measurement Method

**Figure 1:**
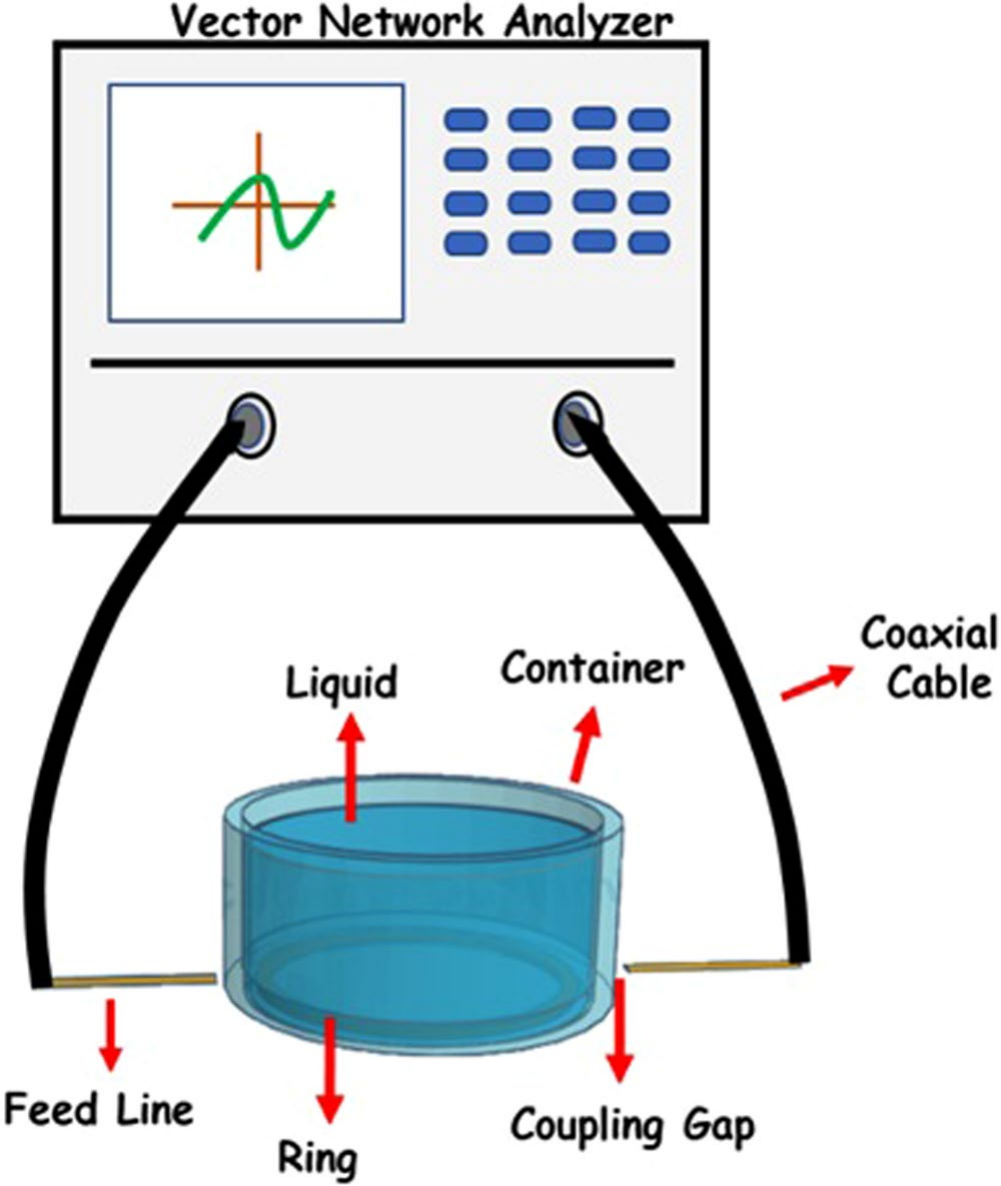
The schematic view of RRM.

Ring resonator is utilized the characterization of materials determining the dielectric properties at microwave frequency range. Besides, it is generally used in microwave devices such as couplers, filters, mixers, oscillators, and antennas. The process of this method can be explained as a relationship between frequency and resonance^26^. A structure of ring resonator method consists of a ring on substrate and transmission line to measure the magnitude of *S*_21_ (dB) and phase values. The characteristic impedance was selected as 50 Ω^27^. The schematic view of this method is shown in Fig. 1 which was drawn by us. There is closed loop, two coupling gaps, and two feed lines as input and output that is contacted to a Vector Network Analyzer (VNA). The measurement system gets its name from the shape of the antenna.

The applying of ring resonator method is easy to use and simple in realization. First time, a microstrip line type of this method was used and afterwards the different types were applied such as coupled lines, coplanar lines, and inverted or suspended microstrip line^28^. The liquids were placed in different vessels. To measure the *S*_21_ parameter at microwave frequencies, a VNA is used in this spectroscopy. It should be considered; the results of RRM must verify with a well-known reference which can be water sample. Thus, the calibration standards are implemented easily to achieve a good accuracy for various liquids. The water dipole losses are sufficiently high at broadband frequency^29^.

The ring antenna resonates when the wavelength of RF energy propagating in the feed line is equivalent to the surrounding of the ring. The transmission and reflection coefficients of material under test in this measurement system by a VNA. The change of resonance according to reference signal measured provide to extract the permittivity of a sample. RRM is able to measure liquids as well as solids. A container was made which can be surrounded the ring and it put over the set-up.

## Analysis Algorithm

Various clustering techniques have been used for many applications. These techniques can be sorted as k-means and principal component analysis, partial least squares, self-organizing maps, and particle swarm optimization algorithms. They can be preferred to cluster the samples with different forms. In this study a well-known and user-friendly classification method was selected to distinguish the liquids. The problem can be defined as determining the k (integer) points in Rd (d-dimensional space), called centers, in n data points for K-means technique. Hence, the distance of mean squared from every data point to nearest center. This process is called squared error distortion. The number of clusters should be pre-specified in data set. The appropriate cluster number is determined by a trial and error process and this is a blind side due to it makes more difficult the clustering process. Therefore, a set might be adopted instead of a single default K. Because, the reflection of specific characteristic of reasonable large data set is very important to achieve a good clustering^19^.

**Figure 2:**
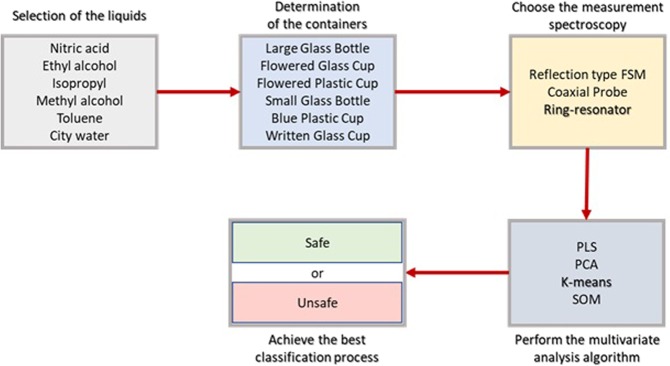
The flowchart of proposed model.

The steps followed to summarize the principle of the proposed model are shown in Fig. 2. First, dangerous liquids are selected, and a well-known water sample is included to contribute to the separation. Interactions of liquids with electromagnetic radiation are observed. The experimental system (RRM), which fast measurement results could be obtained with it, is established to measure the liquids. The collected measurement results (phase and magnitude of *S*_21_) are analyzed by k-means algorithm to achieve the best classification performance.

## Results and Discussion

**Figure 3:**
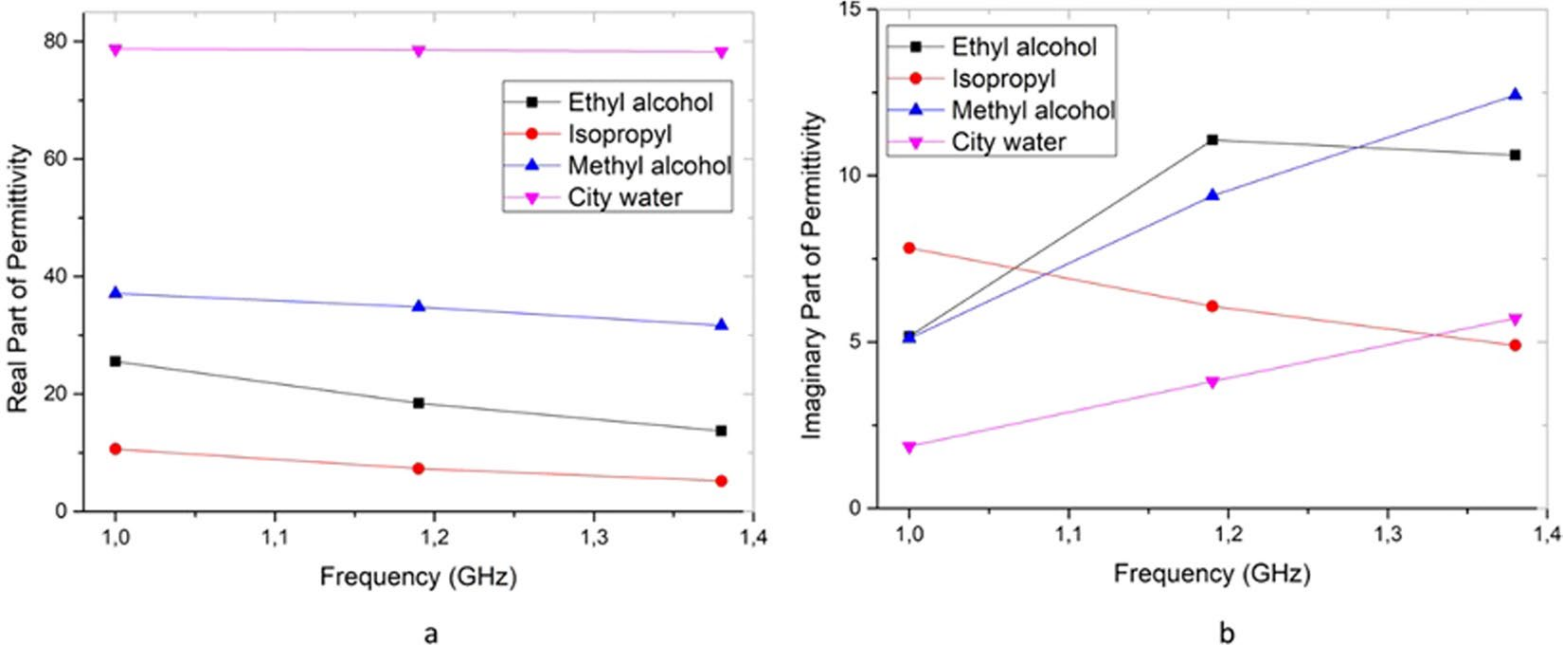
The real and imaginary parts of selected liquids between 1–1.4 GHz.

Three important parameters of a liquid, which are magnitude and phase of *S*_21_, and complex permittivity (*ε*), were measured by using microwave spectroscopy systems. This measurement system is presented as an alternative to ultrasonic method. The attenuation coefficient and propagation velocity parameters can be obtained with ultrasound system, while the transmission (*S*_21_), phase, complex permittivity (ε) parameters can be obtained very easily by microwave spectroscopy systems. Therefore, it is possible to compare the other parameters to be estimated because of the many parameters obtained by the proposed measurement system. Thus, the complex permittivity values (a: real part and b: imaginary part) measured between 1–1.4 GHz by coaxial probe method (CPM) can be analyzed to switch for classification process as seen in Fig. 3. The selected frequency range was determined according to sudden change on transmission parameter (*S*_21_)^30^. In this way, decisive changes that can be used for classification can be processed extremely well by multivariate analysis methods to detect hazardous liquids.

**Figure 4:**
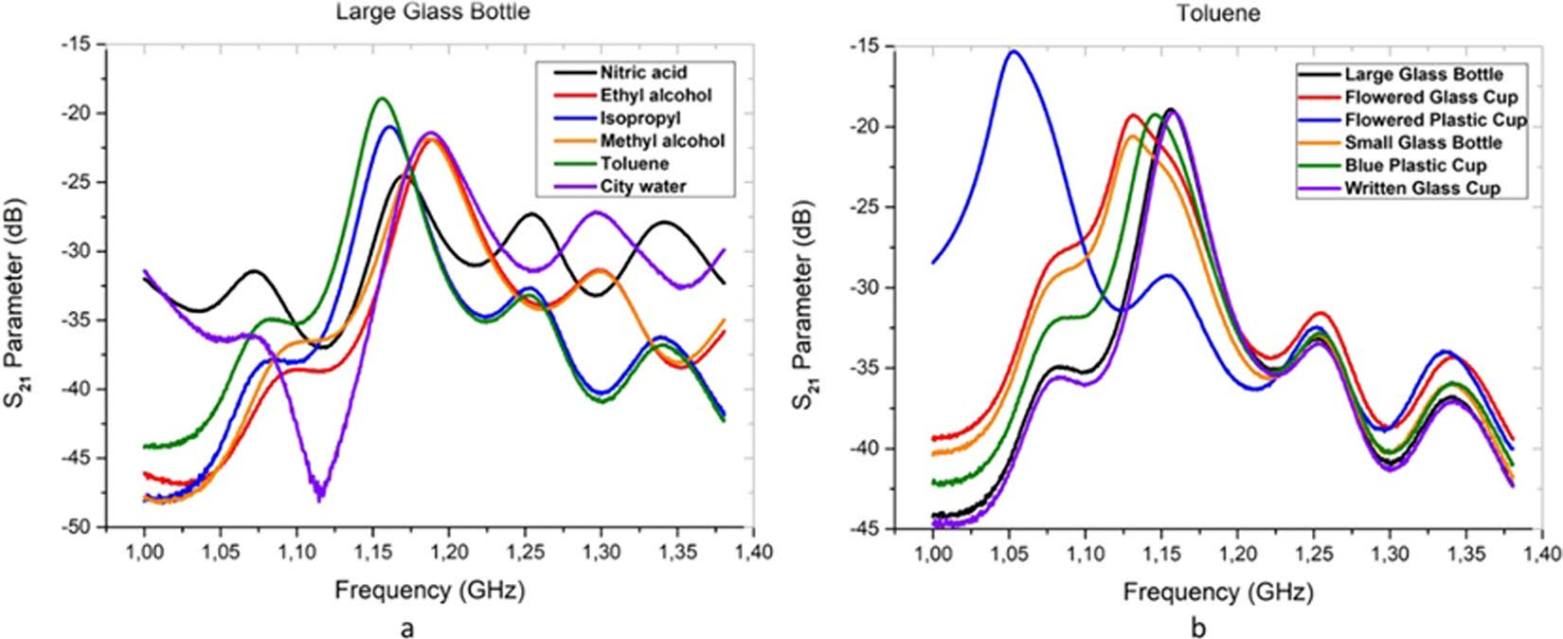
The *S*_21_ parameters of measured liquids between 1–1.4 GHz.

Actually, if it is possible to measure the all liquids in different container types, the CPM can be very appropriate spectroscopy. According to Fig. 3, the permittivity values of safe and unsafe liquids are very different from each other and the permittivity value below 40 can be considered as dangerous. However, while the CPM is a contactless method, RRM is a non-contactless method. Then, the probe of CPM must be touched to extract the permittivity values of liquids. Therefore, this approach has not any capabilities to measure the liquids in closed box or container. Because it is not possible to measure each sample in contact. Alternatively, the RRM system, which can perform measurement process in a short time, facilitates the classification of liquids. As seen in Fig. 4, the magnitude values of liquids were measured by RRM. In Fig. 4a, all liquids were measured in same container (large glass bottle) to indicate the magnitude of *S*_21_ parameter. Nevertheless, the *S*_21_ parameters of toluene sample in six different containers were measured as shown in Fig. 4b.

**Figure 5:**
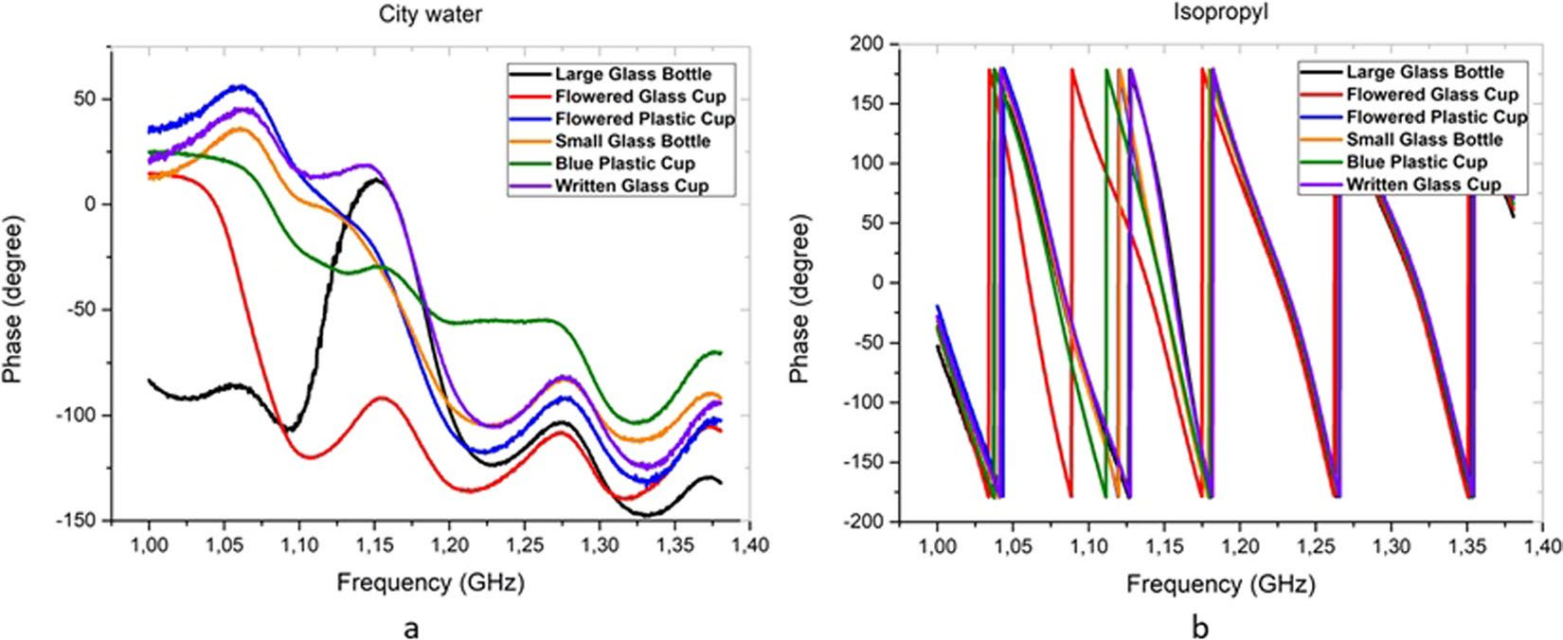
The phase parameters of city water and isopropyl samples between 1–1.4 GHz in all containers.

In the large glass bottle, the transmission parameter (in dB) of 5 different liquids (alcohol group) were showed nearly same reaction to the electromagnetic radiation. However, the reaction of the city water sample is different from the alcohol group. The reactions of toluene were obtained almost the same for every container. However, the classification process was not performed successfully using *S*_21_ parameter. Therefore, instead of *S*_21_ magnitude, the phase parameter is preferred to obtain the cluster groups. Nevertheless, the phase (in degree) parameters were measured to distinguish the liquids as shown in Fig. 5. The reactions of two samples (a: city water and b: isopropyl) to the electromagnetic radiation have different patterns.

**Figure 6:**
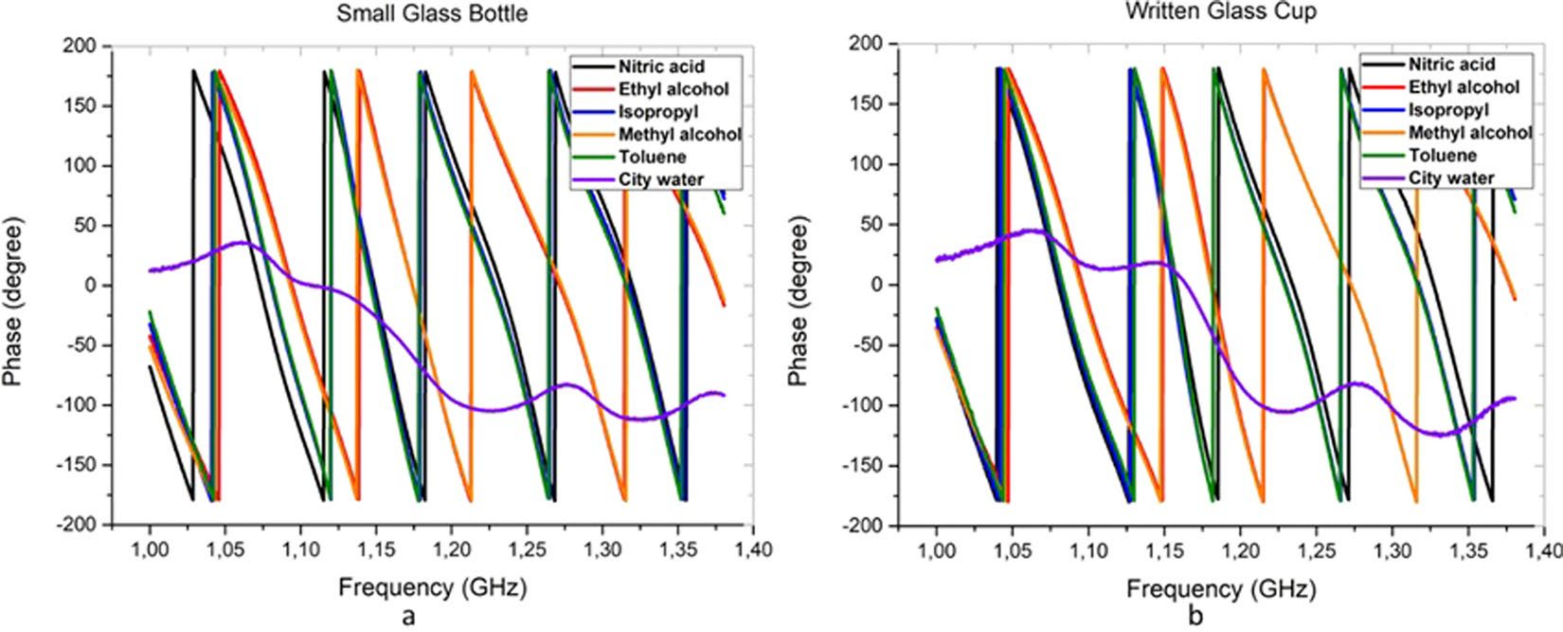
The phase parameters of all liquids between 1–1.4 GHz in two containers.

The phase values of city water are almost same all containers except the large glass bottle. For Isopropyl sample, almost the all patterns are similar to each other, even though a little difference appeared between 1.10–1.15 GHz. The similarities and differences between the phase patterns of liquids can be seen better in Fig. 6.

**Figure 7:**
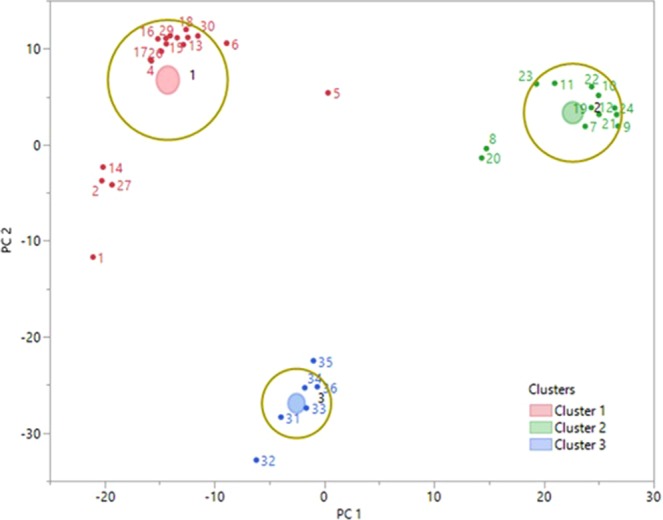
The classification process of liquids by k-means.

In two containers, the phase patterns of city water are different from other liquids (alcohol group). This situation is promising to classify the unsafe liquids. After determining the distinctive property (parameter) for classification, the first distinction process with k-means was obtained as shown in Fig. 7.

**Figure 8:**
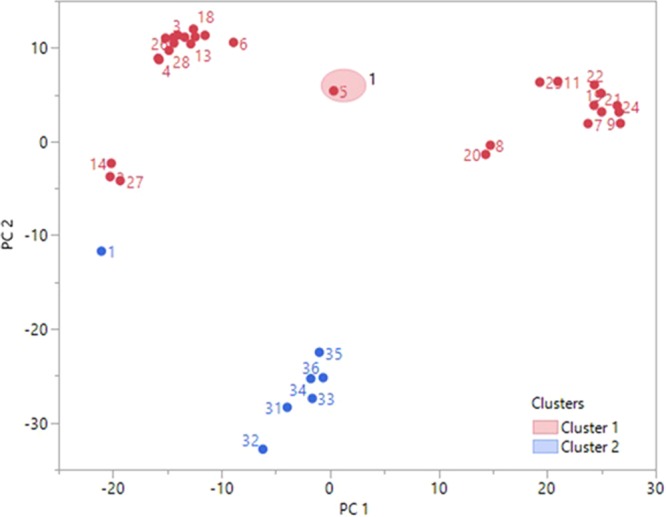
The demonstrating the safe and unsafe groups of measured liquids by k-means.

Three groups were obtained as red, green and blue to demonstrate the capability of classification process. Here, the samples are labeled as follows: Nitric acid (1–6), ethyl alcohol (7–12), isopropyl (13–18), methyl alcohol (19–24), toluene (25–30) and city water (31–36) in all containers. The containers are labeled as follows: Large glass bottle (1, 7, 13, 19, 25, and 31), flowered glass cup (2, 8, 14, 20, 26, and 32), flowered plastic cup (3, 9, 15, 21, 27, and 33) small glass bottle (4, 10, 16, 22, 28, and 34) blue plastic cup (5, 11, 17, 23, 29, and 35) written glass cup (6, 12, 18, 24, 30, and 36) for each samples. As seen in Fig. 7, the blue (city water) group was occurred far from the red and green (alcohols and derivative) groups according to y-axis. In unsafe group, while the nitric acid, isopropyl and toluene samples are together, ethyl alcohol and methyl alcohol are obtained as a separate group. However, it is not clear how the other two groups (red and green) will be qualified. Therefore, after the measurement process, it is necessary to obtain relevant information that a liquid is clearly safe or not. Nonetheless, the k-means algorithm can be expected to divide the all liquid samples (36) into two groups before running. Thus, safe and unsafe groups are obtained by using the phase parameter which is the distinctive feature as seen in Fig. 8.

As seen in Fig. 8, the one sample (number 1) affects the success of the classification obtained with high accuracy. However, it is shown that the material which is dangerous can be detected independently from the container, regardless of which container is used for measuring process. Hence, phase measurement results between 1–1.4 GHz indicate that the difference between containers is not significant. Although the reactions of the alcohol group to electromagnetic radiation are the same in many measurements, some sudden changes due to the containers caused the unsafe group to disperse in the positive and negative direction of the X-axis. However, the distribution observed according to the Y-axis is completely in positive part except one sample (1). This is due to a problem with the capacity of the ring in the RRM regarding the dimensions of the large glass bottle container. This problem may affect the success of the classification process. Therefore, an RRM prototype should be developed that sensitive to the different containers.

The different algorithms can be tried to obtain the best classification results for future studies. Furthermore, the generated database can be tested for different frequency ranges and results of different measurement methods. Moreover, the measured frequency range can be changed to obtain more effective results. However, considering the attenuation of the signal at high frequencies and the adverse effect of ambient conditions, the FSM method should be operated in the mm-wave frequency range to obtain the best result. Also, given the fact that the THz-TDS system provides effective results as fingerprint of material over 300 GHz, it may be a good alternative for classification of liquids.

## Conclusion

In this study, it is tried to show whether the classification can be made independently of the different containers. As a deficiency, the changes of the compiled parameters depending on the temperature have not been investigated. The detection and recognition of the liquid samples precursor components that can be used for production of improvised explosive devices are of extreme importance, particularly in modern security applications such as concert halls, shopping mall and airports. Using the clustering method of k-means algorithm, the liquids examined are successfully classified into the safe and unsafe groups, regardless of the type of a containers used in the study. The selected frequency band between 1–1.4 GHz has given better results for measurement of liquids to obtain the identifier reaction of electromagnetic waves. Besides that, for future studies, a data base can be composed with measured liquids to reduce the process time as well as can be grouped like safe and unsafe.
