# Author Correction: Demonstration of Container Effects on Recognition Process of Liquids Using a Ring-Resonator Measurement Method

**DOI:** 10.1038/s41598-020-62683-8

**Published:** 2020-03-31

**Authors:** Turgut Ozturk

**Affiliations:** Department of Electrical-Electronics Engineering, Bursa Technical University, Bursa, Turkey

Correction to: *Scientific Reports* 10.1038/s41598-019-49102-3, published online 29 August 2019

The Acknowledgements section in this Article was omitted. The Acknowledgements section should read:

“The author thanks İlhami Ünal and Aysun Sayıntı for providing the measurement data made at Marmara Research Center of TUBITAK.”

